# Human Visceral Adipose Tissue Macrophages Are Not Adequately Defined by Standard Methods of Characterization

**DOI:** 10.1155/2019/8124563

**Published:** 2019-01-03

**Authors:** Alecia M. Blaszczak, Anahita Jalilvand, Joey Liu, Valerie P. Wright, Andrew Suzo, Bradley Needleman, Sabrena Noria, William Lafuse, Willa A. Hsueh, David Bradley

**Affiliations:** ^1^Diabetes and Metabolism Research Center, Division of Endocrinology, Diabetes & Metabolism, Department of Internal Medicine, The Ohio State University Wexner Medical Center, Columbus, OH 43210, USA; ^2^Division of General and Gastrointestinal Surgery, Department of Surgery, The Ohio State University Wexner Medical Center, Columbus, OH 43210, USA; ^3^Department of Microbial Infection and Immunity, The Ohio State University Wexner Medical Center, Columbus, OH 43210, USA

## Abstract

Obesity is associated with a state of chronic low-grade inflammation both systemically and within specific tissues, including adipose tissue (AT). In murine models of obesity, there is a shift in the inflammatory profile of the AT immune cells, with an accumulation of proinflammatory M1 macrophages that surround the expanding adipocyte. However, much less is known about the immune cell composition and how to best define AT macrophages in humans. *Objective*. The goals of the current study were to determine the contribution of macrophages to the stromal vascular fraction (SVF) in lean versus obese human visceral AT (VAT); examine the expression of common M1, M2, and pan macrophage markers; and determine the association of specific macrophage types with known biomarkers of obesity-related cardiometabolic disease. *Research Design and Methods*. VAT biopsies were obtained from obese (*n* = 50) and lean (*n* = 8) patients during elective surgery. Adipocytes and SVF were isolated, and the SVF was subjected to flow cytometry analyses. *Results*. Our results indicate that VAT macrophages are increased in obesity and associate with biomarkers of CVD but that many macrophages do not fall into currently defined M1/M2 classification system based on CD206 receptor expression levels. *Conclusions*. VAT macrophages are increased in obese subjects, but the current markers used to define macrophage populations are inadequate to distinguish differences in human obesity. Further studies are needed to delineate the function of AT macrophages in the maintenance and progression of human AT inflammation in obesity.

## 1. Introduction

The prevalence of obesity has risen to epidemic proportions in the last decade, affecting greater than 30% of adults in the United States [1]. In accordance with this increase, there has been a proportional rise in the incidence of obesity-related comorbidities, including type 2 diabetes mellitus (T2DM), atherosclerosis, heart disease, and stroke [2]. The underlying pathophysiologic effects of obesity that lead to these comorbidities are still largely unknown but are fundamentally related to expansion of visceral adipose tissue (VAT) [3, 4]. Given the association between obesity, the AT immunoenvironment, and inflammation [5–7], it is of paramount importance to correctly identify and characterize the immune cell populations in VAT and how they contribute to obesity-related comorbidities.

AT is comprised of adipocytes, the major depot for energy storage, as well as the stromal vascular fraction (SVF), which contains preadipocytes, immune cells, and endothelial cells [8]. One of the primary immune cells, the macrophage, is characterized as a M1, proinflammatory CD11c-expressing macrophage, or a M2, anti-inflammatory CD206-expressing macrophage [9]. The SVF in lean mice is composed of primarily anti-inflammatory immune cells including M2 macrophages [10, 11], regulatory T cells (Tregs) [12, 13], eosinophils, and innate lymphoid type 2 cells (ILC2s) [14]. With chronic overnutrition, as occurs in obesity, the inflammatory milieu of the AT shifts to include a greater abundance of proinflammatory immune cells, such as M1 macrophages [11, 15–17], CD8+ T cells [18], and Th1 T cells [19]. Previous studies have estimated that ~40% of the SVF in mouse AT consists of macrophages, mainly derived from the bone marrow [15] and that these macrophages are recruited via a complicated crosstalk between adipocytes, endothelial cells, and blood monocytes [20]. Murine models that disrupt macrophage expression of CCR2, an important receptor for chemotaxis, demonstrate reduced weight gain, insulin resistance, and liver fat accumulation despite consumption of a high-fat diet (HFD) [21], while transgenic overexpression of monocyte chemoattractant protein 1 (MCP-1) leads to the recruitment of macrophages into AT and the development of insulin resistance [22]. In obese mice, macrophages can also undergo local expansion within AT [23]. While less is known about the role of immune cells in human AT, early studies have shown a similar increase in macrophage infiltration into obese human AT [15, 24], with an increase in M1-like macrophages [4, 25], metabolically activated macrophages [26], and in some cases M2-like macrophages [27], suggesting that the M1 and M2 polarization is overly simplistic [28, 29].

Therefore, the primary goals of this study were to determine the contribution of macrophages to the SVF in obese versus lean human subjects and their relationship to obesity-related cardiometabolic disease and evaluate the utility of the classic M1 and M2 macrophage marker classification in these two patient populations.

## 2. Results

### 2.1. Subject Characteristics

There were no differences in age between the lean and obese subject groups. Body mass index (BMI), as well as multiple metabolic parameters including the presence of diabetes, insulin resistance (HOMA-IR), and fasting insulin, was significantly higher in obese compared to lean patients. Conversely, adiponectin was lower in the obese patients (Table 1). Fourteen patients within the obese group and one patient (treated with metformin) in the lean group had type 2 diabetes; thus, for the measurement of glucose and insulin and the calculation of HOMA-IR, these patients were excluded from analysis.

### 2.2. Macrophages Are Increased in Obese AT but Do Not Follow M1:M2 Polarization

**Figure 1:**
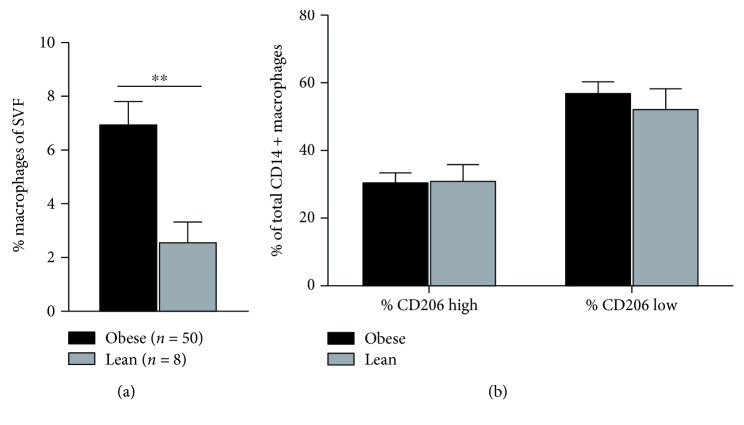
(a) % macrophages of total SVF cells in obese and lean visceral adipose tissue and (b) differential expression of CD206 on adipose tissue macrophages.

**Figure 2:**
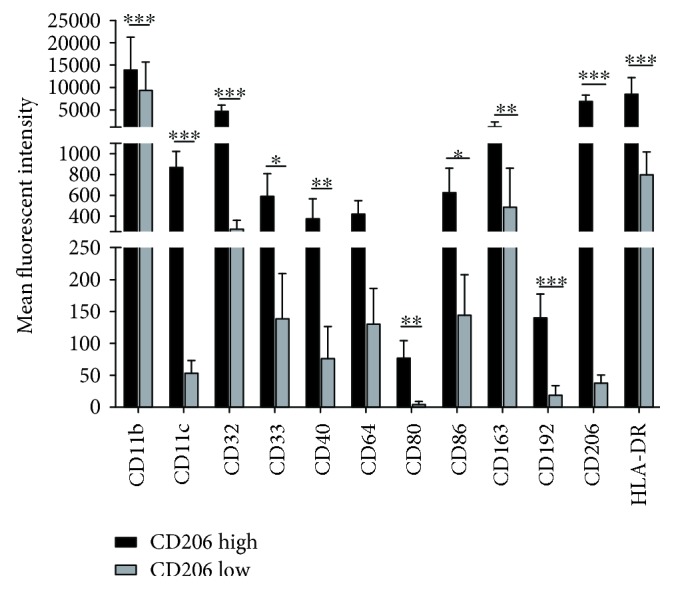
Expression of common monocyte and macrophage markers (CD11b, CD32, CD33, CD64, and CD192) along with M1 (CD11c, CD40, CD80, CD86, and HLA-DR) and M2 (CD163, CD206) surface markers on CD206 high- and low-expressing obese ATMs.

Macrophages as a percent (%) of SVF cells in obese patients were nearly three times that seen in the lean counterparts (7.0 ± 0.8% vs. 2.6 ± 0.7%; *p* = 0.0086) (Figure 1). Patterns of cluster of differentiation (CD)206 expression were similar between both groups, with roughly 30% of AT macrophages expressing high levels of CD206 and around 60% expressing low levels of CD206 (Figure 1). These results indicate that classifying macrophages by CD206 does not adequately distinguish differences in the AT macrophage population observed in lean compared to obese humans. Furthermore, within the population of macrophages with high CD206 expression, there was higher expression of CD11b, CD11c, CD32, CD80, CD86, CD163, CD192, and HLA-DR in obesity, suggesting that these macrophages were activated and shared components of both classical M1 and M2 expression patterns (Figure 2). With only 2 lean patients available for receptor expression analysis, we found that there was some overlap between CD206 high ATM receptor expression between the lean and obese visceral ATMs although there were some striking differences (Figure S3).

### 2.3. AT Macrophages Positively Correlate with Circulating Total Cholesterol

**Figure 3:**
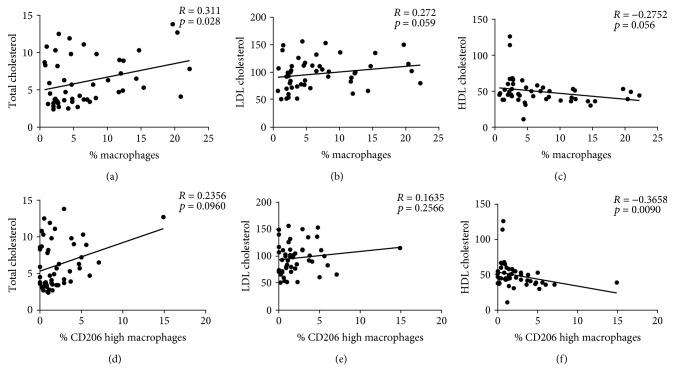
Correlation of %ATMs with (a) total cholesterol, (b) LDL cholesterol, and (c) HDL cholesterol as well as %CD206 high ATMs with (d) total cholesterol, (e) LDL cholesterol, and (f) HDL cholesterol.

For all patients, the proportion of macrophages in visceral AT was positively and significantly associated with total cholesterol concentration. The proportion of AT macrophages also trended towards a positive association with circulating low-density lipoprotein cholesterol (LDL), with a negative relationship with high-density lipoprotein cholesterol (HDL) concentration (Figure 3). When we further examined the CD206 high population of macrophages, we found a significant negative correlation with HDL and a trend for total cholesterol. In contrast, there was no relationship between VAT macrophages or %CD206 high ATMs and insulin resistance (HOMA-IR) (Figure S2).

## 3. Discussion

While it is well known that macrophages are important contributors to the inflammatory microenvironment of AT in murine models of obesity, their role in human AT remains unclear. The goal of this study was to determine (1) the contribution of macrophages to the SVF in human visceral AT, (2) the impact of obesity on AT macrophage distribution and characterization, and (3) the relationship between AT macrophages and the development of risk factors for obesity-related cardiometabolic complications. While we demonstrate that VAT macrophages are increased in obese patients and are related to biomarkers of CV disease risk, the proportion of CD206 high- and low-expressing populations was similar between lean and obese visceral AT. Furthermore, within the obese patients, CD206 high macrophages had increased expression of both M2 markers (CD163) and M1 markers (CD11c, CD80/86, and HLA-DR). While there were only two lean patients for comparison, the CD206 high ATMs showed similar expression patterns to the obese in CD11c, CD80, CD163, CD192, and HLA-DR, whereas others appear to be much lower in the CD206 high ATMs within the lean patients including CD11b, CD32, CD40, CD64, CD86, and CD206 suggesting that there is some difference in receptor expression between the activated ATMs in lean and obese visceral AT.

These cumulative findings suggest that human AT macrophages exhibit a more complex model of macrophage activation than previously described [11] and that the current M1:M2 polarization does not sufficiently identify the differences in human lean and obese subjects.

The increase in macrophage population seen in this study is congruent with murine models of obesity. Several groups have published on the relative contribution of macrophages to the total SVF compartment, with most studies reporting that macrophages are the primary immune cell within AT, comprising about 50% of the total SVF cells [15, 22, 23]. In contrast, we now show that in obese humans, macrophages account for only ~10% of the total SVF cells. This is similar to previous reports suggesting that around 10% of the SVF is considered macrophages in human omental AT [20]. One potential factor contributing to this observation is the difference in circulating T lymphocytes between mouse and human blood. In mice, the lymphocyte is the largest contributor to the circulating immune cell pool [30], with CD8+ T cells as the first responder to HFD feeding. These CD8+ T cells are critical to the subsequent influx of macrophages. In fact, genetic depletion of CD8+ T cells in mice leads to a reduction in AT macrophages and an improvement in insulin sensitivity [18]. In addition, the majority of macrophages within mouse AT arise from the bone marrow and are recruited to the AT under states of chronic overnutrition [20]. In humans, however, the most abundant circulating cell is the neutrophil. Initial studies in human AT suggest that neutrophils contribute to maintenance of the chronic low-grade inflammation seen in obesity [31] but may not provide the same stimulus for macrophage recruitment as CD8+ T cells in mice.

While initial studies in mouse AT suggested the presence of two distinct macrophage populations [11, 17], more recent studies have shown that this model is too simplistic for defining AT macrophages [26]. Furthermore, studies in human AT have shown that macrophages that express anti-inflammatory markers are also capable of promoting AT inflammation [29], while others have shown that macrophages in obesity may express both common M1 and M2 markers [24, 25, 32], CD11c and CD206, respectively. Our data supports this distribution of AT macrophages in the obese state with concurrent expression of CD11c and CD206 but significantly expands upon the existing literature. We found that these cells also express numerous other macrophage markers that were previously unstudied. From our patient cohort, we found 2 distinct populations: CD206 high- and CD206 low-expressing cells. The CD206 high group also expressed significantly higher levels of common macrophage markers including Fc receptors (CD32), complement receptors (CD11b), chemokine receptors (CD192), and scavenger receptors (CD163) as well as molecules important for antigen presentation and immune cell activation (CD40, CD80, CD86, and HLA-DR). Studies on macrophages in human atherosclerotic lesions have shown that expression of CD163, a common M2 marker, is associated with atherosclerotic plaque progression as well as increased vascular permeability, angiogenesis, microvascular inflammation, and immune cell recruitment [33]. This data suggests that not all macrophages expressing common M2 markers are anti-inflammatory, similar to our findings. The second distinct group, CD206 low macrophages, expresses much lower levels of all markers except CD64, which trended towards decreased expression. These cells seem to be similar to what has been described as the M0 or double-negative macrophage in AT [32]. We hypothesize that these cells are resident macrophages in AT that are involved in tissue surveillance. This population of macrophages does not adopt a proinflammatory phenotype and has low expression of the chemokine receptor CD192 (CCR2) which is essential for monocyte chemotaxis into the tissue [21]. As such, these results further highlight the heterogeneity of human AT macrophages and the need for more expansive macrophage delineation.

Finally, our study explored the relationship of macrophage accumulation to the development of biomarkers for obesity-related complications. Previous studies have shown that the secretion of proinflammatory cytokines by macrophages [34] plays a key role in the development and long-term maintenance of insulin resistance [35]. In our patient population, we did not find an association between AT macrophages or CD206 high ATMs and circulating glucose, insulin, or HOMA-IR (Figure S2). This may be due to the small sample size, the use of a less sensitive measure of insulin resistance [36], or the low percentage of macrophages found in human SVF. However, we did observe a relationship between %AT macrophages and circulating total cholesterol, as well as trends for both HDL (negative) and LDL (positive). When we further examined the % of CD206 high ATMs with total cholesterol, HDL, and LDL cholesterol, we found a positive trend for total cholesterol and a significant negative association with HDL. These findings are in part supported by the novel role that macrophages may play in human AT; recent studies have reported that AT macrophages function to buffer lipids from storage in the adipocyte [37]; while most macrophages reside in crown-like structures, others can take up excess lipid and become lipid-laden foam cells, particularly within omental fat [38]. In obesity, these macrophages are unable to adequately buffer excess lipids within the AT, leading to lipid spill over into the circulation [39] and resulting in the development of hyperlipidemia.

There are several limitations of our study including utilizing HOMA-IR as a surrogate model of insulin resistance. In order to better study insulin resistance in this setting and to explore its relation to macrophage number, more direct and sophisticated methods of insulin sensitivity quantification may need to be performed. Furthermore, the impact of many common medications on the inflammatory microenvironment of AT is unknown, and several patients (particularly within our obese cohort) were on systemic therapies for T2DM, hypertension, and hyperlipidemia, which could alter the immune cell profile within AT. Despite these limitations, our study provides compelling evidence for a difference in AT macrophages between lean and obese human visceral AT, as well as novel data regarding macrophage characterization in obesity. Future studies are needed to examine the mechanistic function of human AT macrophages and their recruitment to, and role in sustaining, the inflammatory microenvironment of AT in obesity.

In conclusion, macrophages are increased in VAT of obese patients, associate with CV risk parameters, and contribute to only ~10% of the SVF. However, obese AT macrophages do not demonstrate clear M1:M2 polarization, with a subset of CD206 high AT macrophages showing increased expression of both pro- and anti-inflammatory markers.

## 4. Methods

### 4.1. Patient Selection

Eligible adult patients undergoing surgical intervention were recruited from the Center of Minimally Invasive Surgery at The Ohio State University Wexner Medical Center (OSUWMC). VAT from obese patients was obtained during either Roux-en-Y gastric bypass or sleeve gastrectomy and from lean patients during elective, intra-abdominal surgery, primarily either cholecystectomy or hernia repair. Patients were excluded from the study if they were current smokers, were taking a chronic steroid or anti-inflammatory agent, had end-stage renal or liver disease, or had a past diagnosis of acquired immune deficiency syndrome or neoplastic disease. A total of fifty-eight patients were included in this study (age 21–75 years old, BMI 18–40 kg/m^2^). Demographics, body mass index (BMI), fasting circulating lipids (LDL, HDL, and total cholesterol), and presence of comorbidities were determined at their preoperative visit. All studies were approved by The Ohio State University Institutional Review Board (IRB#H0471). Informed consent was ascertained for all enrolled patients, along with a complete medical evaluation prior to study enrollment.

### 4.2. Study Design and Experimental Procedures

Peripheral blood samples were obtained in the preoperative setting prior to induction of anesthesia. The blood was placed on ice until further processing. Visceral adipose tissue (VAT) biopsies were obtained at the time of surgery in both obese patients undergoing bariatric surgery and lean patients undergoing nonemergent elective surgery. The samples were collected from the operating room and were brought to the laboratory where they were processed within sixty minutes following a collagenase digestion and density centrifugation separation into adipocytes and the SVF as previously described [40, 41].

### 4.3. Quantification of Circulating Insulin, Glucose, and Adipokines

Blood samples were spun down for 10 minutes at 2000 ×*g* to separate the plasma. Plasma was then frozen and stored at −80°C until analysis. For the measurement of insulin (*μ*U/ml), adiponectin (ng/ml), and leptin (ng/ml), ELISA kits were ordered from EMD Millipore (cat #s: EZHI-14K, EZHADP-61K, and EZHL-80SK, respectively) and were completed as per manufacturer's instructions. For the measurement of glucose (mg/dl), a Glucose Liquicolor (Stanbio Laboratory, REF#1070-125) reagent kit was used according to the manufacturer's protocol. The homeostatic model of insulin resistance (HOMA-IR) was used to estimate insulin resistance in these patients and was calculated as

(1) $$\begin{array}{c} HOMA‐IR=\frac{glucose\left(mg/dl\right)*insulin\left(\mu U/ml\right)}{405}, \end{array}$$

### 4.4. Characterization and Quantification of Macrophages

For the phenotypic flow analyses, SVF was resuspended in FACS buffer and incubated with human Fc blocker (Human TruStain FcX, cat #: 422302) for 10 minutes prior to surface staining. The macrophages were identified as CD14+ (BV421, clone: HCD14, cat #: 325628) after exclusion of debris (FSC and SSC) and dead cells (Fixable Viability Dye eFluor 506, eBioscience cat #: 65-0866). They were further characterized by the expression of CD206 (APC, clone: 15-2, cat #: 321110), the mannose receptor, frequently used to differentiate M2 macrophages. Further characterization of macrophages was examined including common macrophage and monocyte markers: CD11b (AF488, clone: ICRF44, cat #: 561687, BD Pharmingen), CD32 (FITC, clone: FUN-2, cat #: 303204), CD33 (PE, clone: WM53, cat #: 303403), CD64 (PE, clone: 10.1, cat #: 305008), and CD192 (FITC, clone: K036C2, cat # 357215), and common M1 markers: CD11c (PerCP-Cy5.5, clone: 3.9, cat #: 301624), CD40 (BV605, clone: 5C3, cat #: 334335), CD80 (PE, clone: 2D10, cat # 305208), CD86 (PE, clone: IT2.2, cat #: 305405), and HLA-DR (BV605, clone: L243, cat #: 307640), as well as common M2 markers: CD163 (BV605, clone: GHI/61, cat #: 333615), and was quantified by mean fluorescent intensity (MFI) within the CD206 low and high populations [42]. For all antibodies listed above, the appropriate isotype controls were run to determine gating and background MFI (Figure S1). All flow antibodies were purchased from BioLegend unless otherwise indicated. The stained samples were run on the BD LSRII through the Flow Cytometry Core in the Comprehensive Cancer Center. All samples were analyzed with FlowJo v7.6.1 (Tree Star) software.

### 4.5. Statistical Analysis

All data is reported as mean ± SEM. For all between group comparisons, since data was not normally distributed, Mann-Whitney *U* tests were performed. To determine associations, Spearman's correlations were performed. Significance was indicated at *p* < 0.05. Analysis was performed using SPSS 22.o for Windows and graphs were generated using GraphPad Prism.

## Figures and Tables

**Table 1:** Patient demographics with all values expressed as mean ± SEM.

	Lean (*n* = 8)	Obese (*n* = 50)
BMI (kg/m^2^)	23.3 ± 0.4	49.2 ± 1.2^∗∗∗^
Age (years)	45 ± 5	42 ± 2
Presence of diabetes	1/8	14/50
Fasting glucose (mg/dL)^#^	79 ± 5	89 ± 3
Fasting insulin (*μ*IU/ml)^#^	2.9 ± 0.9	18.3 ± 2.6^∗∗∗^
HOMA-IR score^#^	0.6 ± 0.2	3.9 ± 0.7^∗∗∗^
Plasma adiponectin (ng/ml)	18360 ± 2895	7908 ± 652^∗∗∗^
Plasma leptin	27.8 ± 8.8	57.3 ± 4.5^∗∗^

^∗^ *p* < 0.05, ^∗∗^ *p* < 0.01, and ^∗∗∗^ *p* < 0.001. BMI: body mass index; HOMA-IR: homeostasis model assessment of insulin resistance. ^#^Excludes patients with diabetes.

## Data Availability

The data used to support the findings of this study are available from the corresponding author upon request.

## Additional Points

*Reprint requests*. David Bradley, MD.

## Supplementary Materials

Supplementary Materials Supplemental Figure 1: the gating structure for all samples can be seen within the panel. Cellular debris was removed by FSC and SSC gating. Then, the dead cells were excluded by eFluor 506 viability dye. In order to capture the macrophage population, cells were gated on CD14. Lastly, cells were gated for CD206 and receptor expression was determined as MFI. Supplemental Figure 2: % adipose tissue macrophages and % CD206 high macrophages were also compared to measures of insulin sensitivity including glucose (*R* = 0.2876, *p* = 0.0991), insulin (*R* = 0.2891, *p* = 0.0921), and HOMA-IR (*R* = 0.224, *p* = 0.2029); % CD206 high glucose (*R* = 0.2584, *p* = 0.1340), insulin (*R* = 0.3153, *p* = 0.0610), and HOMA-IR (*R* = 0.2303, *p* = 0.1831) however, none of the correlations reached statistical significance. For all three correlations, patients with known diabetes were excluded from analysis. Supplemental Figure 3: MFI was calculated for lean and obese AT macrophages while there do appear to be some differences; statistics were not carried out since there were only two subjects in the lean group.
