# Supplementary material for: Human Visceral Adipose Tissue Macrophages Are Not Adequately Defined by Standard Methods of Characterization
Source: J Diabetes Res. 2019 Jan 3;2019:8124563. doi: 10.1155/2019/8124563 (PMC6335856; doi:10.1155/2019/8124563)
Supplement: Supplementary Materials — Supplemental Figure 1: the gating structure for all samples can be seen within the panel. Cellular debris was removed by FSC and SSC gating. Then, the dead cells were excluded by eFluor 506 viability dye. In order to capture the macrophage population, cells were gated on CD14. Lastly, cells were gated for CD206 and receptor expression was determined as MFI. Supplemental Figure 2: % adipose tissue macrophages and % CD206 high macrophages were also compared to measures of insulin sensitivity including glucose (R = 0.2876, p = 0.0991), insulin (R = 0.2891, p = 0.0921), and HOMA-IR (R = 0.224, p = 0.2029); % CD206 high glucose (R = 0.2584, p = 0.1340), insulin (R = 0.3153, p = 0.0610), and HOMA-IR (R = 0.2303, p = 0.1831) however, none of the correlations reached statistical significance. For all three correlations, patients with known diabetes were excluded from analysis. Supplemental Figure 3: MFI was calculated for lean and obese AT macrophages while there do appear to be some differences; statistics were not carried out since there were only two subjects in the lean group. [file 8124563.f1.pdf]

Supplemental Figures

The gating structure for all samples can be seen below (Figure S1). Cellular debris was removed using the FSC and SSC gating. Then the dead cells were excluded by eFluor506 viability dye. In order to capture the macrophage population, cells were gated on CD14. Lastly cells were gated for CD206 and receptor expression was determined.

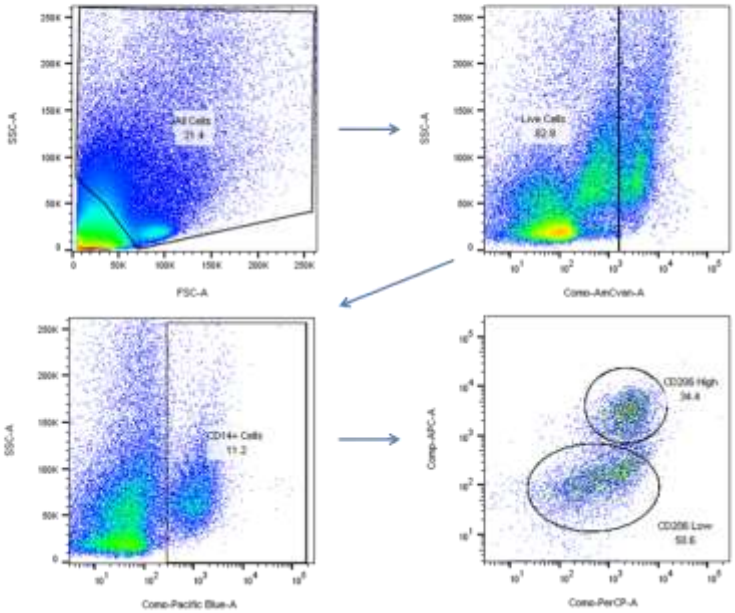

Figure S1: Representative flow gating scheme for human ATMs

%ATM as well as %CD206 high ATMs were also compared to measures of insulin sensitivity including glucose, insulin and HOMA-IR (Figure S2); however, none of the correlations reached statistical significance.

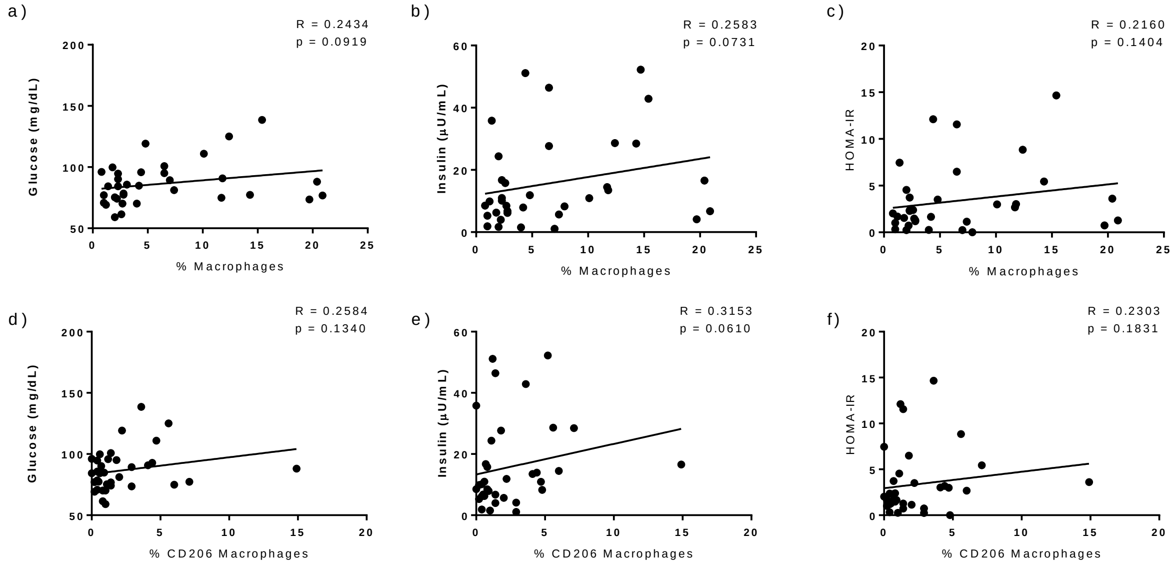

Figure S2: Correlation of % ATMs with a) glucose (mg/dL), b) insulin (µU/mL) and c) HOMA-IR as well as % CD206 High ATMs with d) glucose (mg/dL), e) insulin (µU/mL) and f) HOMA-IR excluding patients with diabetes.

Lastly, we also looked at receptor expression of all receptors in CD206 high expressing cells in lean and obese patients (Figure S3). Since there were only 2 patients in the lean group, statistics were not done. Based on the patterns below, there does appear to be some difference in the lean and obese ATM receptor expression in the CD206 high populations.

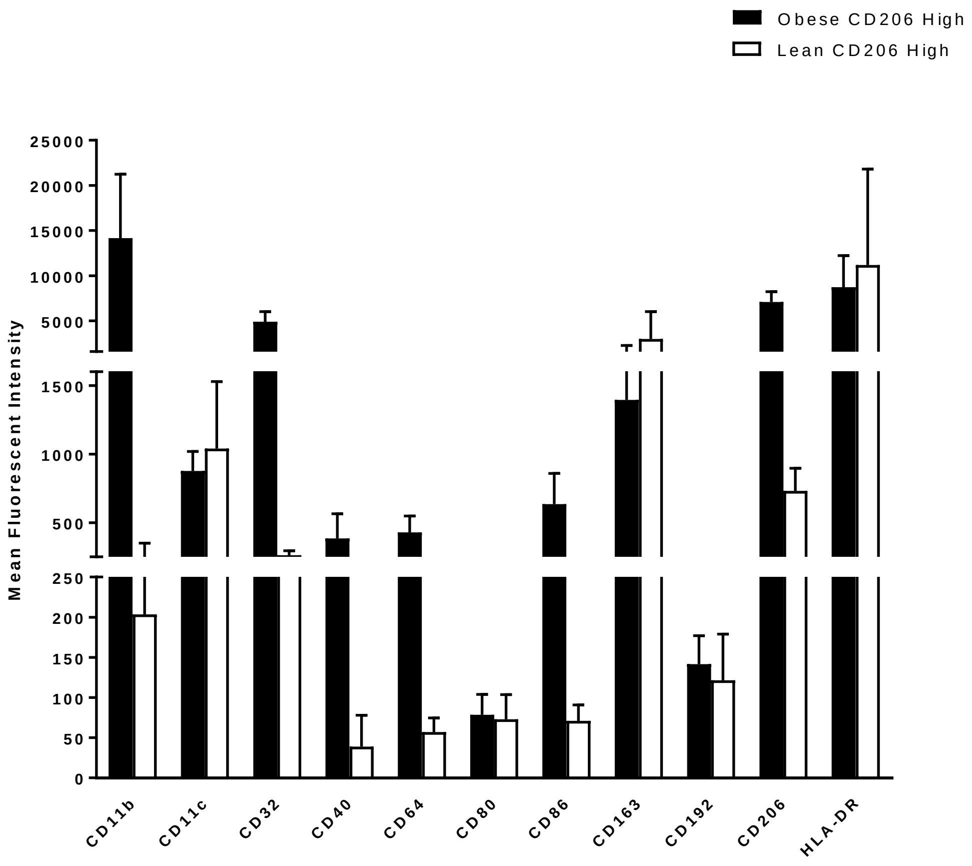

Figure S3: Expression of M1, M2 and metabolically activated surface markers on CD206 high expressing ATMs in obese (n = 27) and lean (n = 2) patients
